# Development of a framework to guide research into policies promoting physical activity and healthy diets in the European context: the system-based Policy Evaluation Network (PEN) framework

**DOI:** 10.1093/eurpub/ckac068

**Published:** 2022-11-29

**Authors:** Carlijn B M Kamphuis, Sarah Forberger, Nanna Lien, Eva Rehfuess, Aleksandra Luszczynska

**Affiliations:** Department of Interdisciplinary Social Science, Utrecht University, Utrecht, The Netherlands; Leibniz Institute for Prevention Research and Epidemiology—BIPS, Bremen, Germany; Department of Nutrition, Institute of Basic Medical Sciences, University of Oslo, Oslo, Norway; Institute for Medical Information Processing, Biometry and Epidemiology, LMU Munich, Munich, Germany; Pettenkofer School of Public Health, Munich, Germany; Wroclaw Faculty of Psychology, SWPS University of Social Sciences & Humanities, Wroclaw, Poland; Melbourne Centre for Behavior Change, Melbourne School of Psychological Sciences, University of Melbourne, Melbourne, Australia

## Abstract

**Background:**

The Policy Evaluation Network (PEN) is a multidisciplinary Pan-European research consortium focussing on policies affecting dietary intake, physical activity and sedentary behaviour. At the start, the PEN consortium expressed the need for an overarching, system-based framework covering the complexities between the different domains of the policy process (design, implementation and outcomes) in order to execute all research activities in a coherent way. This article describes the PEN framework itself and its development process.

**Methods:**

A staged approach to the development of a system-based framework was executed between February 2019 and February 2022. We started with a point-of-departure framework, made use of existing models, collected PEN outputs at different project stages (through online meetings, e-mail exchanges and workshops with PEN researchers) and drew updated versions of the framework, which resulted in the system-based PEN framework.

**Results:**

The system-based PEN framework depicts the policy process as a complex system, visualizing the dynamic interrelations between and within policy domains (i.e. policy design, policy implementation and policy outcomes), the ways they interact with the context, and how to assure a focus on equity in each domain.

**Conclusions:**

The system-based PEN framework may guide researchers and professionals involved in the evaluation of health- or sustainability-related policies to consider their evaluation in a comprehensive picture, including domain interactions, contextual influences and equity considerations, as these can have important implications for the scope of their research. The stage-based process as applied for the development of the PEN framework can serve as a template for other research projects wishing to develop their own framework.

## Introduction

Unhealthy dietary intakes, lack of physical activity (PA) and extensive sedentary behaviour are important risk factors for major, non-communicable diseases, and often more prevalent in vulnerable populations (e.g. people in low socioeconomic position), which leads to considerable health inequalities.^1^ Evidence is needed on which specific policies aiming to promote PA and healthy diets work best, for whom, in which contexts, what the underlying mechanisms are, and which policies will contribute to a reduction in health inequalities. To generate this evidence, rigorous scientific evaluations of the implementation and impact of policies are needed, but comprehensive studies across Europe are rare. In response to this gap, 28 research institutes from seven European countries and New Zealand combined their expertise and established the Policy Evaluation Network (PEN). PEN pursued a multidisciplinary research programme, organized in seven work packages, aiming to advance tools to benchmark, implement and evaluate policies directly or indirectly affecting dietary intake, PA and sedentary behaviour in Europe, as well as to understand how policies increase or decrease health inequalities.^2^

Policy includes a set of interrelated decisions concerning the selection of goals and a set of actions, i.e. the means of achieving them within a certain context.^3^^,^^4^ Decisions, actions and contextual characteristics interact, which may shift their focus over time within a dynamic policy process. Three broad domains as part of the policy process are: policy design, policy implementation and policy outcomes.^5^ While policy implementation and policy outcomes are at the core of PEN, PEN also aims to improve the understanding of the complex interrelations and feedback loops between policy design, implementation and outcomes, for instance how outcomes can inform future policy design. Further, PEN aims to better understand why specific policies work in some contexts and not in others—given that contexts may differ widely between countries involved in PEN. Further, PEN asks how and why policy effects differ between different population groups (e.g. lower and higher socioeconomic groups). To achieve these aims and carry out all research activities of the seven work packages in a coherent way, an overarching framework was needed.

A myriad of frameworks and theories, each with its own lens on the policy process, could be of potential relevance to PEN. Recent overviews of generic policy theories and frameworks discuss the usefulness of the Multiple Streams Framework, the Punctuated Equilibrium Theory, the Narrative Policy Framework, the Advocacy Coalition Framework, the Policy Feedback Theory, the Diffusion of Innovation Theory and the Institutional Analyses and Development Framework.^6–8^ Additionally, there are frameworks that are concerned with specific policies, e.g. the System Framework on PA.^9^ Another group of frameworks seek to aid with selecting or developing health policy options and provide relevant criteria, such as human rights, sociocultural acceptability and societal implications in the WHO-INTEGRATE framework.^10^ However, none of these existing approaches included what was needed for PEN: a framework covering the complexities between the different domains of the policy process (design, implementation and outcomes), with a focus on context and equity.

In this article, we describe our stage-based approach towards the framework development process and the resulting system-based PEN framework. Our motivations for doing so are twofold. First, the system-based PEN framework can help future studies specifically focussing on either content, implementation or impact evaluation to zoom out and consider the full policy process, as interactions between domains as well as contextual influences and equity considerations will have important implications for the scope of their own research. Second, the development process may in itself serve as a template for other complex research projects aiming to develop their own framework and integrate new project insights throughout the project.

## Methods: development of the PEN framework

A ‘core team’ of PEN researchers (i.e. the authors of this article) led the development of the framework. A larger ‘Theory Group’, consisting of PEN researchers representing all work packages, provided feedback and input during the various stages of the framework’s development (names and expertise listed in the Supplementary file).

### Step 1: Choosing a point-of-departure framework and development approach

**Figure 1:**
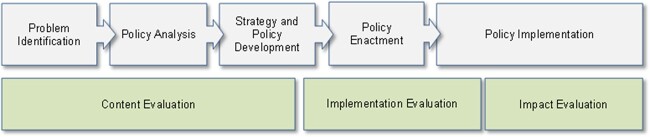
CDC framework used by PEN as a point-of-departure framework (reprinted with permission from Centers for Disease Control and Prevention, 2013)^5^

During the research proposal preparation, the Centres for Disease Control and Prevention (CDC) Framework for Policy Evaluation had been chosen as conceptual framework (see figure 1).^5^ The CDC framework was considered useful as it was developed specifically for health policy purposes and depicts the full policy process.^5^ However, the CDC framework assumes a linear sequence of actions with one-way effects (e.g. from policy design to implementation to outcomes), which did not match with PEN’s focus on interactions and feedback loops. We decided that, taking the CDC framework as a ‘point-of-departure’, we would develop a PEN framework following existing procedures for logic model development.^11^^,^^12^

A logic model is described as ‘…—a graphic description of a system—… designed to identify important elements and relationships within that system’.^13^ Basic logic models in programme evaluation systematically present the relationships between available resources or inputs; planned activities; outputs and desired outcomes and impact.^11^ System-based logic models (similar to ‘program theories’ or ‘causal logical models’) explicitly recognize the complex interplay (or links) between as well as within the distinct domains included in the model, with the many links often being multi-directional in nature.^9^^,^^11^ The associations between the domains of the system may result in changes reverberating throughout the system. System-based logic models can be developed in three distinct ways.^12^ ‘A priori models’ are chosen during the research planning phase and remain unchanged throughout the research process. ‘Iterative models’ evolve throughout the research process, often in a relatively uncontrolled manner. ‘Staged models’ start off with an initial model and pre-specify time points in the research process at which a new version of the logic model can be specified, thereby allowing controlled incorporation of knowledge emerging during the research process. A staged approach^12^ to the development of the system-based PEN framework was chosen to allow new project outputs to be included in the framework at pre-specified time points. The staged approach to the development of the PEN framework is presented in table 1.

**Table 1:** Stages of the development of the system-based PEN framework

Stage of framework	Research insights included	Delivered
The linear CDC-based point-of-departure framework (figure 1)	PEN proposal	February 2019 (start of the PEN project)
The initial system-based PEN framework (Supplementary file)	Literature review of existing frameworks; workshop of the Theory Group during the PEN kick-off meeting	November 2019 (9 months into the project)
The interim system-based PEN framework (Supplementary file)	Finding from PEN project collected among PEN researchers in summer and autumn 2021	November 2021 (33 months into the project)
The final system-based PEN framework (figure 2)	Workshop of the Theory Group in January 2022	February 2022 (end of the PEN project)

### Step 2: Developing the initial system-based PEN framework

The initial PEN framework (see Supplementary file) was finalized in November 2019. The domains, constructs, context characteristics and associations included in the initial framework were derived from the CDC framework,^5^ the system-based logic model template^11^ and the Context and Implementation of Complex Interventions (CICI) framework.^3^ In addition, public policy approaches were used to define overarching concepts within the system approach focussing on actor constellations (principal-agent), power/interests/political agenda, role of jurisdiction, level of analyses, administrative capacity and resources.^14^ The initial PEN framework was developed by the core team and drafts were shaped by feedback from the Theory Group based on the current status of their work packages and their general expertise.

### Step 3: Collecting input for revision of the initial framework

During the second half of 2021, the core group had online meetings and e-mail exchanges with Theory Group members as well as other PEN researchers to collect input for revision of the initial framework. Researchers commented that the linear arrangement of the boxes representing policy design, policy implementation and policy outcomes did not match the systems perspective. Therefore, the three boxes were rearranged into a circle, more clearly depicting how the domains feed into each other. Further, the figure was found to be too crowded with arrows depicting interactions, but lacked a recognition of the important role of co-occurring policies. Therefore, we reduced the number of arrows to the most essential ones, and mentioned interactions with other co-occurring policies in the three main boxes. Most other feedback concerned information to be added to a textual description of the figure rather than changes to the figure itself (see Supplementary file). The interim system-based PEN framework, i.e. a revised version of the initial framework, is also available in the Supplementary file.

### Step 4: Collecting feedback for revision of the interim framework

In January 2022, a workshop was held to discuss the interim framework with Theory Group members. A rich variety of feedback was collected regarding the figure itself (e.g. the content of the three boxes), and the textual description accompanying the figure (e.g. a description of the figure itself, as well as examples of PEN results to illustrate this) (see Supplementary file).

## Results: the final system-based PEN framework

**Figure 2:**
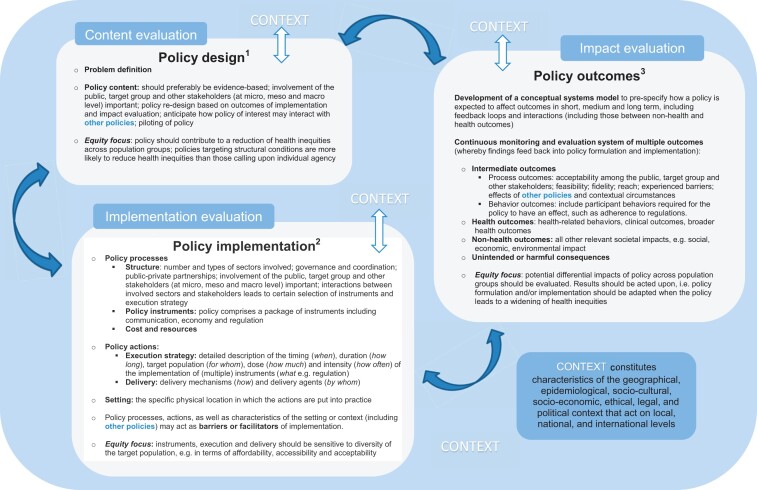
The system-based PEN framework ^1^Policy design starts with formatting a precise definition of the problem that should be tackled, and then, the solution to that problem, i.e. the policy content. Involvement of the public and stakeholders (at micro, meso and macro level) is important to increase acceptability and support for the policy. Piloting the policy is an important step in policy design. To decrease health inequities, structural actions (e.g. reducing the sodium content of highly processed foods) are preferred over actions that call on individual agency (e.g. putting information about sodium content on food labels).^20^ ^2^Regarding policy implementation, we distinguish between ‘policy processes’, ‘policy actions’ and the ‘setting’ in which the policy is implemented (adapted from Rohwer et al. 2017).^11^ ‘Policy processes’ include ‘structural aspects’, ‘policy instrument selection’ (i.e. a package of instruments including communication, economy and regulation,^22^ e.g. communication about the new policy via media, combined with infrastructural changes and regulations) and ‘costs and resources’ (e.g. financial aspects, but also knowledge of instruments and the best execution strategy). ‘Policy actions’ concern the ‘execution strategy’ and ‘delivery’. The ‘setting’ is the specific physical location in which the actions are put into practice. ^3^Regarding policy outcomes, it is important to evaluate intermediate outcomes, health outcomes, non-health outcomes as well as unintended or harmful consequences. ‘Health outcomes’ include health-related behaviours (like dietary intakes and PA levels), clinical outcomes (e.g. morbidity and mortality) and broader outcomes (such as well-being, life expectancy and quality of life). ‘Non-health outcomes’ refer to all other relevant societal impacts of a policy, e.g. social, economic, environmental outcomes (e.g. cycling infrastructure and healthy food availability) or sustainability-related outcomes (e.g. water and energy use of food production and fair payment). ‘Unintended or harmful consequences’ outcomes may arise as well, for instance due to interactions with other policies co-occurring at the same time.

The final system-based PEN framework (figure 2) includes three main boxes representing the domains of policy design, policy implementation and policy outcomes. Bidirectional arrows indicate that complex associations are assumed between the three domains, as well as between each of the domains and the context they are embedded within. Associations may include first-order mechanisms, with the use of policy instruments (implementation) leading to behaviour change of individuals, groups or structures (outcomes), and second-order mechanisms (feedback processes), with policy outcomes affecting further instrument choices or execution strategies.^14^ How equity should be taken into account in each of the three domains is described in the respective boxes in figure 2. Equity refers to the absence of avoidable, unfair or remediable differences among groups of people, whether those groups are defined socially, economically, demographically or geographically or by other means of stratification.^15^ Below, we give a textual description of how the system-based PEN framework as visualized in figure 2 should be ‘read’, illustrated by results of the PEN project.

### Policy design

The domain of policy design comprises the ‘problem definition’ and the ‘policy content’. The policy content depends on how the problem is exactly defined and framed, which in turn depends on the stakeholders involved in the discussion (e.g. representatives of the target population, lobbyists and policymakers) and the overall context. The initiation of policy design is often driven by a specific event, a ‘window of opportunity’, or a tipping point that gives rise to the policy’s inception. Whether policymakers are willing to support a given policy depends on their subjective beliefs about the effectiveness, potential side effects, appropriateness and likely public acceptability of that policy.^16^

Policy content and re-design should preferably be evidence-based, i.e. based on the results of studies evaluating the implementation and impact of earlier versions of the policy. However, successful knowledge translation and evidence use requires long-term engagement, relationship building, and effective communication between researchers, policymakers and other stakeholders, illustrating how interactions between the involved stakeholders at micro, meso and macro levels affect policy design. Policy content for policies to promote healthy dietary intakes and PA can be found in the Food-Epi Framework^17^ and the PA-Epi framework,^18^ which include best-practice examples of policies.

Already during policy design, potential (unwanted) side effects of policies (as addressed in the policy outcomes domain) need to be considered, as this allows to re-shape the policy or develop additional, counteracting policies. Likewise, interactions with other, existing policies should be kept in mind. Furthermore, with a view to a later policy outcome evaluation, it is essential to identify critical health and health behaviours as well as non-health outcomes (including potential determinants of health and health-behaviours at the policy, environmental and organizational level); for some of these outcomes, indicators may already be available in European health surveillance systems.^19^

Regarding equity, it is important to realize that policies targeting structural conditions are more likely to reduce health inequities than those calling upon individual agency.^20^ Preferably, a selection of mutually reinforcing actions should be considered during policy design, i.e. actions that simultaneously affect several mechanisms and determinants leading to less favourable health and health-behaviours among vulnerable population groups, including the wider determinants of health inequities.^21^

### Policy implementation

In the domain of policy implementation, we distinguish between ‘policy processes’, ‘policy actions’ and the ‘setting’ in which the policy is implemented (figure 2).^11^ ‘Policy processes’ include ‘structural aspects’ (e.g. interactions between involved stakeholders and across sectors), ‘policy instrument selection’ (i.e. communication instruments, such as mass media campaigns, economic instruments, such as taxation, and regulation instruments),^22^ and ‘costs and resources’ (i.e. financial costs, but also knowledge about instruments and the best execution strategies). ‘Policy actions’ concern the ‘execution strategy’ and ‘delivery’. The ‘setting’ is the specific physical location in which the actions are put into practice.

Any of the specific characteristics mentioned as part of policy processes, policy actions or setting can act as barriers or facilitators for the policy implementation and should be evaluated.^23^ PEN findings regarding implementation processes identified the following implementation barriers and facilitators as most important: cost, networking with other organizations/communities, external policies, structural characteristics of the setting, implementation climate, readiness for implementation and knowledge/beliefs of involved individuals.^24^ We also found that knowledge on how certain policies are actually implemented, for instance the implementation of a sugar tax, is limited.

Further, many policy implementation frameworks (including CICI,^3^ DPAS—schools^25^ and DPAS—general^26^) suggest bidirectional associations between implementation process variables and the implementation content. For example, if stakeholders who implement the policy evaluate the implementation process as not fitting with the needs of the target population, or observe that the policy of interest (being implemented) is not compatible with other policies (already implemented), they may propose an adjustments of policy content or targets. To be pro-equity, implementation actions should be designed in such a way (e.g. bottom up, in co-creation with target groups) that they match with preferences, capabilities and opportunities of (different) target groups.

### Policy outcomes

The policy outcome domain comprises ‘intermediate outcomes’, ‘health outcomes’, ‘non-health outcomes’ and ‘unintended or harmful consequences’, defined according to a ‘conceptual systems model’ and assessed through ‘monitoring and evaluation systems’. To understand which data are needed for impact evaluation, and to foresee potential unintended consequences, it is important to develop a conceptual systems model early on in the policy process to pre-specify how a policy is supposed to impact on which outcomes and within which time frames (short, medium and long term), including feedback loops and interactions. Evaluation of policy outcomes is not a one-time endeavour; instead, continuous surveillance and monitoring is necessary and findings should lead to an adaptation of the policy (policy re-design) or the implementation strategy.

Diverse types of evidence and study designs should be considered for evaluating policy impact (i.e. holistic sense-making, using evidence from different study designs and drawing on theoretical insights as well as empirical evidence). This is important to prevent a bias in the evidence base, as it is easier to conduct traditionally ‘gold-standard’ evaluations of certain types of policies (e.g. food pricing) than others (e.g. food promotion).^27^ For the evaluation of policy outcomes in different contexts (e.g. different cities and different European countries), the harmonized collection of comparable data on multiple outcomes is required.^28^ Finally, the types of data needed to evaluate the policy effects should be thought through, for instance data collection among individuals (e.g. often at high cost, requiring active participation, such as when asking participants to report their exercise levels) vs. data collection on a population level (e.g. often at lower cost, requiring no active participation, such as when observing the usage of public areas for exercise).

A focus on equity during impact evaluations means that potential differential effects of policies for different population groups (e.g. groups of higher or lower socioeconomic status, or those with different ethnic backgrounds) are investigated, as well as their underlying mechanisms. It is important to evaluate how potential adverse consequences of a policy could unequally impact some population groups, and results should be acted upon in case the policy leads to a widening of inequalities, e.g. by policy re-design, additional policies or a different implementation strategy.

### Interactions between policy domains and with the context

Interactions between policy domains, i.e. how one domain affects the other two, should be considered at any stage of the policy process. Examples of interactions include: when impact evaluations (policy outcome domain) show that the public acceptability of selected policy actions is low, this may imply that adaptations to the execution strategy (e.g. enforcement strategies) or delivery mechanisms or agents are needed (policy implementation domain). Or: when scientific evidence generated by impact evaluations point to a high impact of certain policies (policy outcome domain), this is important information for future policies (policy design domain), in order to make policies more evidence-based. PEN studies showed that the GRADE Evidence to Decision framework was helpful to make the process of developing recommendations for future policy design based on scientific evidence more systematic, transparent and comprehensible.^29^

Policy domains not only interact with each other, but also with the context. Context reflects ‘a set of characteristics and circumstances that shapes, interacts and modifies policy design, implementation and outcomes’.^3^ The CICI framework^3^ was applied to specify seven important contexts: geographical, epidemiological, sociocultural, socioeconomic, ethical, legal and political characteristics. The context also includes other existing policies that may affect the design, implementation and outcomes of the policy of interest (examples of interactions with other policies are mentioned in figure 2). A PEN review concluded that the political context, sociocultural context and socioeconomic context (resources) are particularly important for the implementation domain.^30^ PEN studies on the implementation of the European School Fruit and Vegetable schemes in European countries pointed towards similar characteristics: economic resources and governance structure (both the legal and political aspects) at the macro (national) level and the sociocultural context at the meso (school) level.^31^ Studies in which the Food-Epi framework was applied to assess and benchmark the level of implementation of food environmental policies in different European countries^32^ and Europe^33^ came across an illustrative example of the role of the legal context: what was legally possible in different countries in terms of, for instance, food reformulation depended on European as well as national jurisdiction.

PEN studies on the implementation of Sustainability Urban Mobility Plans (SUMPs) showed a clear example of the importance of the historical/political context: the extent to which local governments in different European cities had a historical tradition in applying principles of sustainability or ‘Health in All Policies’ in their policies appeared to impact SUMP development and implementation.^34^ Other examples of important political contextual influences, for instance when it comes to implementing a sugar tax, included: the policy cycle in a certain country, political systems and capacity, confidence in political institutions and lobby forces.^35^ The importance of the political context aligns with frameworks like the Multiple Streams Model,^36^ that especially stress the importance of the political context (‘policy action and climate’), and how that interacts with problem identification and policy design (‘making policy choices’), in creating ‘windows of opportunity’ for policy changes and implementation.

### Involvement of stakeholders

Stakeholders include the target population, implementers, policymakers, lobbying groups and all others that have an opinion on, are somehow involved in, or affected by the policy. Different stakeholders have different views and interests, e.g. on the importance of a policy, and whether/how it should be implemented and evaluated. The different stakeholders involved and their interactions will affect policy design, implementation and outcomes. A special emphasis should be placed on interprofessional and intersectoral collaboration and participatory approaches. The involvement of the target population in each domain is crucial.

## Discussion

The system-based PEN framework provides an overview of the complex processes of policy design, implementation and outcomes, how each of these feed into each other and interact with the context, and how a focus on equity can be assured in each domain. The framework builds on a broad range of underlying theories and concepts as well as the specific insights of the PEN project and has been developed through a staged approach, guided by a system-based logic model template.^11^ Equipped with this overview, researchers and practitioners have a flexible map of key concepts for examining a given policy. For a multidisciplinary project like PEN, having a framework that captures the full policy process while acknowledging complexity and a systems perspective was of utmost importance.

Besides its value for the current project, the PEN framework as well as the described development process may also be of value beyond PEN. The system-based PEN framework can help future studies that focus on either content, implementation or impact evaluation to zoom out and consider cross-domain interactions, contextual influences and equity considerations, since these will have important implications for the scope of their research. Further, the framework development process, involving all work package leaders and other key PEN researchers, helped to make a large project like ours more coherent. The described stage-based development process could serve as a template for other large research projects wishing to develop their own framework, allowing for the integration of new project insights throughout the project. Also, although the system-based PEN framework was developed to guide the evaluations of diet- or PA-related policies, the framework is flexible and lends itself to new applications, e.g. to other health-promoting policies or sustainability-related policies. To increase the likelihood of future use, the framework will be actively promoted among researchers as well as policymakers, e.g. via symposia, websites, social media and targeted distribution within the professional networks of PEN researchers.

Researchers focussing on one specific domain (e.g. implementation), can use the PEN framework in combination with more specific frameworks. For instance, within PEN, other frameworks applied included the Food-EPI framework,^17^ the DEDIPAC Surveillance and Monitoring framework,^28^ the GRADE Evidence to Decision framework,^29^ the Consolidated Framework for Implementation Research framework,^23^ frameworks for understanding health inequities (like the WHO Health Equity Policy Tool)^15^ as well as frameworks taking a systems perspective (like the Action Scales model).^37^ A PEN review^38^ identified three policy implementation frameworks as particularly comprehensive and useful (i.e. the CICI framework,^3^ the DPAS-general framework^26^ and the DPAS-school framework^25^), as these addressed determinants, processes and outcomes of implementation; included system-, community- and individual-level characteristics; provided information on interlinkages between implementation variables; and included at least some equity factors.

Over the last two decades, research in the fields of dietary intake and PA often had a focus on understanding how ‘environmental’ determinants (such as accessibility and affordability of healthy foods, or availability of infrastructure for active transport) impact on these behaviours, in order to inform policy design. However, as a result of the growing recognition of systems thinking, researchers have started to apply a broader perspective on how behaviours, and policies seeking to affect these, interact within food ‘systems’ and transport ‘systems’.^9^^,^^39^ Systems thinking helps to examine the factors determining a problem, the relations between these factors and changes over time; it views actions as integrated and interacting across political, social, cultural and economic domains.^9^ This broadened perspective is important for health policy evaluation and lends itself to bringing in the views of other sectors and of sustainability-related outcomes (fair payment, effects of water and energy use for food production on climate). We hope the system-based PEN framework will be of use to future studies aiming to evaluate policy design, implementation and outcomes from such a broad societal perspective.

## Supplementary data

Supplementary data are available at *EURPUB* online.

## Supplementary Material

ckac068_Supplementary_Data

## References

[1] Lim SS , Vos T, Flaxman AD, et al A comparative risk assessment of burden of disease and injury attributable to 67 risk factors and risk factor clusters in 21 regions, 1990-2010: a systematic analysis for the Global Burden of Disease Study 2010. Lancet 2012;380:2224–60. 23245609 10.1016/S0140-6736(12)61766-8 PMC4156511

[2] Lakerveld J , Woods C, Hebestreit A, et al Advancing the evidence base for public policies impacting on dietary behaviour, physical activity and sedentary behaviour in Europe: the Policy Evaluation Network promoting a multidisciplinary approach. Food Policy 2020;96:101873.

[3] Pfadenhauer LM , Gerhardus A, Mozygemba K, et al Making sense of complexity in context and implementation: the Context and Implementation of Complex Interventions (CICI) framework. Implement Sci 2017;12:21. 28202031 10.1186/s13012-017-0552-5 PMC5312531

[4] Jenkins WI. Policy Analysis: A Political and Organisational Perspective. London: Martin Robertson, 1978.

[5] Centers for Disease Control and Prevention. CDC’s Policy Brief 1: Overview of Policy Evaluation. 2013. Available at: https://www.cdc.gov/injury/pdfs/policy/brief%201-a.pdf (22 January 2022, date last accessed).

[6] Allen LJ. From multiple streams to muddling through: policy process theories and “Field of Vision”. Open Pol Sci 2020;3:117–27.

[7] Heikkila T , Cairney P. Comparison of theories of the policy process. In: Sabatier P, Weible C, editors. Theories of the Policy Process, 4th edn. Boulder, CO: Westview Press, 2017: 363–89.

[8] Van der Heijden J , Kuhlmann J, Lindquist E, Wellstead A. Have policy process scholars embrace causal mechanisms? A review of the five popular frameworks. Public Policy Adm 2021;36:163–86.

[9] Rutter H , Cavill N, Bauman A, Bull F. Systems approaches to global and national physical activity plans. Bull World Health Organ 2019;97:162–5. 30728623 10.2471/BLT.18.220533 PMC6357559

[10] Rehfuess EA , Stratil JM, Scheel IB, et al The WHO-INTEGRATE evidence to decision framework version 1.0: integrating WHO norms and values and a complexity perspective. BMJ Glob Health 2019;4:e000844. 10.1136/bmjgh-2018-000844 PMC6350705 30775012

[11] Rohwer A , Pfadenhauer L, Burns J, et al Series: clinical epidemiology in South Africa. Paper 3: logic models help make sense of complexity in systematic reviews and health technology assessments. J Clin Epidemiol 2017;83:37–47. 27498377 10.1016/j.jclinepi.2016.06.012

[12] Rehfuess EA , Booth A, Brereton L, et al Towards a taxonomy of logic models in systematic reviews and health technology assessments: a priori, staged, and iterative approaches. Res Synth Methods 2018;9:13–24. 28677339 10.1002/jrsm.1254

[13] Anderson LM , Petticrew M, Rehfuess E, et al Using logic models to capture complexity in systematic reviews. Res Synth Methods 2011;2:33–42. 26061598 10.1002/jrsm.32

[14] Capano G , Howlett M. Causal logics and mechanisms in policy design: how and why adopting a mechanistic perspective can improve policy design. Public Policy Adm 2021;36:141–62.

[15] World Health Organization. Health Equity Policy Tool. A Framework to Track Policies for Increasing Health Equity in the WHO European Region. Available at: https://www.euro.who.int/en/health-topics/health-determinants/social-determinants/publications/2019/health-equity-policy-tool-2019 (22 January 2022, date last accessed).

[16] Eykelenboom M , van Stralen MM, Olthof MR, et al; PEN Consortium. Political and public acceptability of a sugar-sweetened beverages tax: a mixed-method systematic review and meta-analysis. Int J Behav Nutr Phys Act 2019;16:78. 31484538 10.1186/s12966-019-0843-0 PMC6727579

[17] Swinburn B , Vandevijvere S, Kraak V, et al; INFORMAS. Monitoring and benchmarking government policies and actions to improve the healthiness of food environments: a proposed Government Healthy Food Environment Policy Index. Obes Rev 2013;14 Suppl 1:24–37. 24074208 10.1111/obr.12073

[18] Woods C , Volf K, Kelly L, et al The first steps to benchmarking PA policy: the development of a comprehensive physical activity environment policy index (PA-EPI). Eur J Public Health 2022.

[19] Garnica Rosas L , Mensink GBM, Finger JD, et al; PEN Consortium. Selection of key indicators for European policy monitoring and surveillance for dietary behaviour, physical activity and sedentary behaviour. Int J Behav Nutr Phys Act 2021;18:48. 33794923 10.1186/s12966-021-01111-0 PMC8015190

[20] Backholer K , Beauchamp A, Ball K, et al A framework for evaluating the impact of obesity prevention strategies on socioeconomic inequalities in weight. Am J Public Health 2014;104:e43–50. 10.2105/AJPH.2014.302066 PMC4167106 25121810

[21] Djojosoeparto SK , Kamphuis CBM, Harrington JM, et al How theory can help to understand the potential impact of food environment policies on socioeconomic inequalities in diet: an application of Bourdieu’s Capital Theory and the Scarcity Theory. Eur J Public Health 2022. 10.1093/eurpub/ckac052 PMC9706114 36444101

[22] Bemelmans-Videc M-L , Rist RC, Vedung EO. Carrots, Sticks & Sermons: Policy Instruments and Their Evaluation. New Brunswick, USA and London, UK: Transaction Publishers, 2011.

[23] Damschroder LJ , Aron DC, Keith RE, et al Fostering implementation of health services research findings into practice: a consolidated framework for advancing implementation science. Implement Sci 2009;4:50. 19664226 10.1186/1748-5908-4-50 PMC2736161

[24] Lobczowska K , Banik A, Brukalo K, et al Meta-review of implementation determinants for policies promoting healthy diet and physically active lifestyle: application of the Consolidated Framework for Implementation Research. Implement Sci 2022;17:2. 34991624 10.1186/s13012-021-01176-2 PMC8734337

[25] World Health Organization. School Policy Framework: Implementation of the WHO Global Strategy on Diet, Physical Activity and Health. Available at: https://www.who.int/dietphysicalactivity/SPF-en-2008.pdf?ua=1 (8 February 2020, date last accessed).

[26] World Health Organization. Global Strategy on Diet, Physical Activity and Health: A Framework to Monitor and Evaluate Implementation. World Health Organization. Available at: https://www.who.int/dietphysicalactivity/Indicators%20English.pdf (8 February 2020, date last accessed).

[27] Løvhaug AL , Granheim SI, Djojosoeparto SK, et al The potential of food environment policies to reduce socioeconomic inequalities in diets and to improve healthy diets among lower socioeconomic groups: an umbrella review. BMC Public Health 2022;22:433. 35246074 10.1186/s12889-022-12827-4 PMC8895543

[28] Hebestreit A , Thumann B, Wolters M, et al; DEDIPAC Consortium. Road map towards a harmonized pan-European surveillance of obesity-related lifestyle behaviours and their determinants in children and adolescents. Int J Public Health 2019;64:615–23. 30888434 10.1007/s00038-019-01227-y

[29] Schwingshackl L , Zähringer J, Stratil JM, et al Manual for making evidence-based recommendations for health policies and evaluating policies using the “GRADE Approach”. Eur J Public Health 2022.

[30] Lobczowska K , Banik A, Forberger S, et al; on behalf of Policy Evaluation Network (PEN) Consortium. Social, economic, political, and geographical context that counts: meta-review of implementation determinants for policies promoting healthy diet and physical activity. BMC Public Health 2022;22:1055. 35619065 10.1186/s12889-022-13340-4 PMC9137101

[31] Zolfaghari M , Meshkovska B, Lien N; on behalf of the PEN consortium. Applying a system dynamics lens to understand the mechanisms of the European School Fruit and Vegetable Scheme. Eur J Public Health 2022. 10.1093/eurpub/ckac054 PMC9706111 36444113

[32] von Philipsborn P , Geffert K, Klinger C, et al Nutrition policies in Germany: a systematic assessment with the Food Environment Policy Index. Public Health Nutr 2021;25:1691–700. 34881689 10.1017/S1368980021004742 PMC9991688

[33] Djojosoeparto SK , Kamphuis CBM, Vandevijvere S, et al Strength of EU-level food environment policies and policy recommendations to create healthy food environments in EU Member States. Eur J Public Health 2022 (in press). 10.1093/eurpub/ckac010 PMC9159309 35265982

[34] Okraszewska R , Peters NV, Reisch LA, et al Implementation process of sustainable urban mobility plans on physical activity: evidence from Danish, German, and Polish cities. Eur J Public Health 2022. 10.1093/eurpub/ckac069 PMC9706112 36444103

[35] Eykelenboom M , Djojosoeparto SK, van Stralen MM, et al Stakeholder views on taxation of sugar-sweetened beverages and its adoption in the Netherlands. Health Promot Int 2021;37:daab114. 10.1093/heapro/daab114 PMC9053456 34333638

[36] Kingdon JW. Agendas, Alternatives and Public Policies. New York: Wesley, Longman, 1995.

[37] Nobles JD , Radley D, Mytton OT; The Whole Systems Obesity programme team. The Action Scales Model: a conceptual tool to identify key points for action within complex adaptive systems. Perspect Public Health 2021;175791392110067. 10.1177/17579139211006747 PMC9720704 33998333

[38] Lobczowska K , Banik A, Romaniuk P, et al Frameworks for implementation of policies promoting healthy nutrition and physically active lifestyle: systematic review. Int J Behav Nutr Phys Act 2022;19:16. Available at: https://journals.sagepub.com/doi/pdf/10.1177/17579139211006747. 35151330 10.1186/s12966-021-01242-4 PMC8841124

[39] Sawyer ADM , van Lenthe F, Kamphuis CBM, et al; PEN Consortium. Dynamics of the complex food environment underlying dietary intake in low-income groups: a systems map of associations extracted from a systematic umbrella literature review. Int J Behav Nutr Phys Act 2021;18:96. 34256794 10.1186/s12966-021-01164-1 PMC8276221

