# Supplementary material for: Development of a framework to guide research into policies promoting physical activity and healthy diets in the European context: the system-based Policy Evaluation Network (PEN) framework
Source: Eur J Public Health. 2022 Nov 29;32(Suppl 4):iv3–9. doi: 10.1093/eurpub/ckac068 (PMC9706121; doi:10.1093/eurpub/ckac068)
Supplement: ckac068_Supplementary_Data [file ckac068_supplementary_data.docx]

**Online Supplementary File**

**“Development of a framework to guide research into policies promoting physical activity and healthy diets in the European context: The system-based Policy Evaluation Network (PEN) framework”**

(corresponding author: Carlijn Kamphuis, [c.b.m.kamphuis@uu.nl](mailto:c.b.m.kamphuis@uu.nl))

This supplementary file includes five Appendices:

- Appendix 1: Members of the PEN Theory group
- Appendix 2: The initial system-based PEN framework
- Appendix 3: Main feedback on the initial system-based PEN framework, received from PEN researchers via online meetings and e-mail exchanges over summer and autumn 2021
- Appendix 4: The interim system-based PEN framework
- Appendix 5: Main feedback on the interim system-based PEN framework, received from PEN researchers during an online workshop in January 2022

**Appendix 1 Members of the PEN Theory Group**

| **Core team** | **Area of expertise** |
| --- | --- |
| Sarah Forberger, Leibniz Institute for Prevention Research and Epidemiology, BIPS, Germany | Implementation Research and Policy Analysis |
| Carlijn Kamphuis, Utrecht University, Netherlands | Socioeconomic inequalities in health and health-behaviours (PA and diet); food environment; systems thinking |
| Nanna Lien, University of Oslo, Institute of Basic Medical Sciences, Department of Nutrition, Norway | Public health nutrition |
| Aleksandra Luszczynska, SWPS University of Social Sciences & Humanities, Poland | Health psychology; implementation science |
| Eva Rehfuess, LMU Ludwig Maximilians Universität München /Technische Universität München, Germany | Methods for evidence-based public health; evaluation of complex interventions; global public health |
| **PEN researchers** |  |
| Wolfgang Ahrens, Leibniz Institute for Prevention Research and Epidemiology - BIPS, Germany | Epidemiological methods; exposure assessment; health promotion |
| Sara Capacci, UNIBO University, Bologna, Italy | Public policy evaluation; quasi-experimental methods for impact evaluations |
| Janas Harrington, Centre for Health and Diet Research, School of Public Health, University College Cork, Ireland | Public Health Nutrition, Food Environments, Food Policy |
| Antje Hebestreit, Leibniz Institute for Prevention Research and Epidemiology - BIPS, Germany | Harmonization; Europe; surveillance; dietary behavior, physical activity, key indicators. |
| Jeroen Lakerveld, Amsterdam UMC, VU University Amsterdam, Netherlands | Upstream determinants of lifestyle behaviours and chronic disease risk |
| Frank van Lenthe, Erasmus University Medical Center Rotterdam, Netherlands; Department of Human Geography and Spatial Planning, Utrecht University, the Netherlands | Socioeconomic inequalities in health and health-behaviours (PA and diet); urban food environment; systems thinking |
| Mario Mazzocchi, UNIBO University Bologna, Italy | Policy evaluation; food policy; consumer behaviour; statistics for marketing and consumer research |
| Biljana Meshkovska, University of Oslo, Institute of Basic Medical Sciences, Department of Nutrition, Norway | Political Science, Implementation Science, qualitative research |
| Celine Murrin, UCD-PH University College Dublin, Ireland | Public health nutrition |
| Peter Philipsborn, LMU Ludwig Maximilians Universität München /Technische Universität München, Germany | Public health nutrition; food systems; policy analysis; implementation research |
| Juergen Steinacker, UULM, Ulm University, Division of Sport and Rehabilitation Medicine, Ulm, Germany | Population based prevention, physical activity, rehabilitation, internal medicine and cardiology |
| Janine Wendt, UULM, Ulm University, Division of Sport and Rehabilitation Medicine, Ulm, Germany | Public health, prevention, physical activity, diet |
| Catherine Woods, Physical Activity for Health  Research Cluster, Health Research Institute, University of Limerick, Ireland | Physical activity policy evaluation and development; determinants of physical activity |
| Hajo Zeeb, Leibniz Institute for Prevention Research and Epidemiology, BIPS, Germany | Social determinants of health; evaluation of public health interventions. |

**Appendix 2. The initial system-based PEN framework**: integration of the CDC model (CDC 2013), the system-based logic model template (Rohwer et al., 2017), the CICI framework (Pfadenhauer et al, 2017), and public policy approaches (Capano & Howlett, 2019; Howlett, 2019).

**Policy formulation and design**

- **Policy content** (i.e. initial design, and, later in time, potential re-design, depending on outcomes of implementation and effect evaluation)
- **Equity focus** (policy takes into account systematic health differences across population groups and incorporates actions to reduce these)

**Content evaluation**

**Implementation evaluation**

**CONTEXT**

**CONTEXT**

**CONTEXT**

**CONTEXT**

**CONTEXT**

**CONTEXT**

**CONTEXT**

**CONTEXT**

**Impact evaluation**

**CONTEXT**

References

**Implementation**

- **Policy processes**
  - **Structure**: number and types of sectors involved, governance and coordination, public-private partnerships
  - **Policy instruments selected:** policies comprise of packages of instruments and actions in areas of technology, infrastructure, education, laws, and regulations
  - **Cost and resources**
- **Policy actions:**
  - **Execution strategy:** detailed description of the timing (when?), duration (how long?), target population (for whom?), dose (how much?) and intensity (how often?) of the actions (what? e.g. infrastructure, regulation) that arise from the policy.
  - **Delivery:** delivery mechanisms (how?) and delivery agents (by whom?)
  - **Setting (*where)*:** refers to the specific physical location in which the actions are put into practice, including other policies implemented in the same time frame. Policy actions, execution and their delivery interact with the specific characteristics of the setting and the seven contextual domains (see box below)
  - **Equity focus** (execution and delivery sensitive to diversity of the target population, e.g. in terms of affordability, accessibility and acceptability of policy actions)

Anderson, L. M., Petticrew, M., Rehfuess, E., Armstrong, R., Ueffing, E., Baker, P., Francis, D., & Tugwell, P. (2011). Using logic models to capture complexity in systematic reviews. *Research Synthesis Methods*, 2(1), 33-42. https://doi.org/10.1002/jrsm.32

Capano, G., Howlett, M. (2019.) Causal logics and mechanisms in policy design: How and why adopting a mechanistic perspective can improve policy design. *Public Policy and Administration*. [https://doi.org/10.1177/0952076719827068](https://doi.org/10.1177%2F0952076719827068)

Centers for Disease Control and Prevention. CDC’s Policy Policy brief 1: Overview of policy evaluation. 2013b. Available at: <https://www.cdc.gov/injury/pdfs/policy/brief%201-a.pdf> (accessed on November 28th, 2017; policy briefs 1-6, available at this web address were consulted as part of this application)

Centers for Disease Control and Prevention. CDC’s Policy Analytical Framework. Atlanta, GA: Centers for Disease Control and Prevention, US Department of Health and Human Services. 2013a. Available from: <https://www.cdc.gov/policy/analysis/process/docs/cdcpolicyanalyticalframework.pdf>

**CONTEXT constitutes 7 characteristics: the** **geographical, epidemiological, socio-cultural, socio-economic, ethical, legal, and political context characteristics that act on local, national, and international levels. Context includes other policies occurring in a similar time frame as the policy of interest. The context and its characteristics are associated with policy development, implementation and outcomes.**

**FEEDBACK: covering first order (directly affect actor or system behavior) and second order (activating policy learning, diffusion, knowledge transfer, positive & negative) mechanisms; role of temporal dimension**

**Policy outcomes (short, medium and long term)**

- **Intermediate outcomes**
  - Process outcomes: can be quantitative or qualitative, including participation, implementation, acceptability, feasibility, fidelity, reach, barriers experienced, experiences of participants and intervention providers, and effects of other co-occurring policies and contextual circumstances.
  - Behavior outcomes: include participant behaviors required for the policy to have an effect, such as adherence, but can also refer to other behavioral outcomes occurring intentionally or unintentionally.
- **Health outcomes**: health-related behaviors, morbidity, mortality, well-being, and quality of life.
- **Non-health outcomes:** all other relevant impacts of an intervention, e.g. social, economic, or environmental outcomes (e.g. improved air quality).
- **Unintended or harmful consequences**
- **Equity focus** (differential impacts across diverse groups; policy should contribute toward reducing systematic health differences across population groups)

**Appendix 3:** **Main feedback on the initial system-based PEN framework, received from PEN researchers via online meetings and e-mail exchanges over summer and autumn 2021**

*General feedback*

- The linear arrangement of the three main boxes in the figure does not match the systems perspective.
- The figure is too crowded with text and with arrows depicting potential interactions, which makes the graphic difficult to grasp and led to confusion
- Describe that the PEN framework is meant to provide researchers and policy makers with an overview of the full policy process, how different stages/domains affect and interact, and what are important things to keep in mind, e.g. interactions and feedback loops, equity aspects and the important role of the context. Describe potential interactions in more detail and give examples.
- When studies zoom in into one specific domain (e.g. implementation), more specific frameworks can be applied. Give examples of frameworks they used in their own work packages in addition to the PEN framework. Mention that CFIR framework could be helpful to apply in implementation evaluations.
- Explain how ‘equity’ is defined.
- Mention importance of actor/stakeholder involvement and involvement of the target group in all domains. Actors in micro- (social actors), meso- (socio-political actors) and macro-level (politicial). Describe that stakeholder interaction within and between policy domains is important.
- Describe that, within each domain (policy formulation, implementation, impact) different stakeholders have different views and interests, e.g. on the importance of a certain policy, and whether and how it should be implemented and evaluated.

*Feedback regarding the Policy design domain*

- Describe that policy initiation is driven by a specific driver, event or tipping point that gave rise to the policy’s inception.
- Describe that policies should preferably be evidence-based to ensure adequate efficiency of the intervention. However, given the skewed evidence, it should be considered just one input into the policy development process. Further, the translation of scientific evidence requires effective communication of researchers with policy-makers and stakeholders, so that they will not only understand the evidence lying behind proposed actions (to become policy), but they will also understand the need to formulate and implement a given type of policy.
- Mention importance of ‘piloting’ in the text.
- Describe that potential side effects of policies need to be thought through during policy design (as then maybe additional, counteracting policies can be designed as well).
- Describe that as policy content is being created it is essential to already identify the potential indicators of change at different levels (not just on individual outcomes, also ‘ecological’ outcomes).
- Note that structural actions are more likely to reduce inequities than those calling upon individual agency. Consider that mutual reinforcing actions may be needed to tackle health inequities.
- Describe that work within PEN WP6.1 showed that subjective beliefs of policy makers about the effectiveness, potential side effects and appropriateness of a policy intervention play a role in policy development. And also beliefs of policy makers of what the public would think of a certain policy. If policy makers think that the policy would not be seen as acceptable by the public, then they may be less likely to choose for this policy, as they want to be elected during the next elections.

*Feedback regarding the Policy implementation domain*

- Mention that a PEN review study identified 3 frameworks that addressed determinants, processes and outcomes of implementation, included system-, community-, and individual – level characteristics, provided information about the ways the implementation variables are associated and included at least some equity factors. These were: the Context and Implementation of Complex Interventions (CICI) framework; the DPAS (Global Strategy on Diet, Physical Activity and Health") School Policy Framework; and Global Strategy on Diet, Physical Activity and Health: A framework to monitor and evaluate implementation DPAS.
- Mention that many models of policy implementation (including CICI, DPAS - schools, DPAS - general) suggest bidirectorial associations between implementation process variables and the implementation content and bidirectorial variables between effectiveness of policies and their implementation (in particular sustainable implementation)
- Explain that policy actions should be implemented in such a way that these fit with preferences of different groups. So not only evaluate whether actions were perceived as acceptable afterwards, but also in the implementation phase, pro-actively design the implementation strategy in such a way that it matches with preferences/ capabilities/ opportunities of different groups.

*Feedback regarding the Policy outcomes domain*

- Mention that causal loop diagrams or other conceptual systems models are needed for the implementation and impact evaluation of each specific policy
- Give examples of indicators to measure intermediate outcomes, health outcomes and non-health outcomes
- Mention that the loop going from the policy outcome domain to the policy design domain is especially important.
- During impact evaluation studies, data for analysing potential mechanisms should be collected as well, not only final health outcomes.
- Interactions of the policy of interest with other policies should be evaluated.
- Draw on diverse types of evidence when evaluating the impact of a policy in a complex system (i.e. holistic sense-making, using evidence from different study designs and drawing on theoretical as well as empirical evidence). This acknowledges that it is easier to conduct traditionally ‘gold-standard’ evaluations of certain types of policies (e.g. labelling or pricing vs promotion), leading to a bias in the evidence base.
- Potential differential effects of policies for different population groups (e.g. low and high SES groups, or those with different ethnic backgrounds) should be investigated, as well as the potential underlying mechanisms.
- Mention that it is important to consider potential adverse consequences of the policy (especially those unequally impacting some population groups), and how these could be mitigated.
- And potential side effects need to be monitored and evaluated.

*Feedback regarding Context*

- Detail what is meant with ‘context’, and provide concrete examples of contextual influences;
- Highlight that three aspects of the context are particularly important in relation to the implementation domain, namely economic resources, sociocultural context, and political context.
- Mention political climate and the features of political rivalry as examples of the political context; as well as social trust, especially confidence in political institutions.
- Describe the role of the context in more detail, e.g. how historical and cultural traditions affect the development of policy plans (e.g. to what extent principles of sustainability or Health in All Policies are applied)

**Appendix 4: The interim system-based PEN framework**

**CONTEXT**

**CONTEXT**

**
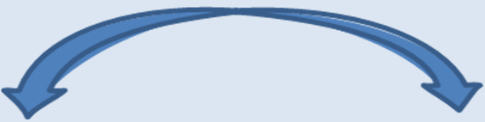
**

**Content evaluation**

**
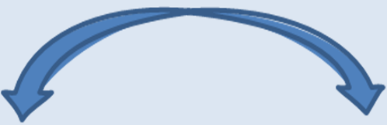

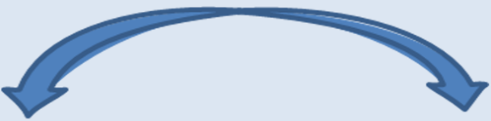
**

**Policy implementation**

- **Policy processes**
  - - **Structure**: number and types of sectors involved, governance and coordination, public-private partnerships; stakeholder involvement; **interactions** between involved sectors and stakeholders may lead to a certain selection of instruments, and certain execution strategy
    - **Policy instruments:** policy comprises of package of instruments in areas of education (e.g. communication via media), technology, infrastructure, laws, and regulations (e.g. conditional or unconditional prohibitions, economic incentives or disincentives)
    - **Cost and resources** (e.g. knowledge of instruments and execution strategy)
- **Policy actions:**
  - - **Execution strategy:** detailed description of the timing (*when*), duration (*how long*), target population (*for whom*), dose (*how much*) and intensity (*how often*) of the implementation of (multiple) instruments (*what* e.g. regulation)
    - **Delivery:** delivery mechanisms (*how*) and delivery agents (*by whom*)
  - **Setting:** the specific physical location in which the actions are put into practice, including **other policies** implemented in the same time frame. Policy processes (e.g. selected instruments) and policy actions (e.g. execution and delivery) **interact** with specific characteristics of the setting and with the seven contextual domains
  - ***Equity focus*** : instruments, execution and delivery should be sensitive to diversity of the target population, e.g. in terms of affordability, accessibility and acceptability

**Policy outcomes (short, medium and long term)**

- **Intermediate outcomes**
  - - Process outcomes: can be quantitative or qualitative, including participation, implementation, acceptability among target group and policy makers, feasibility, fidelity, reach, barriers experienced, experiences of participants and intervention providers, and effects of **other policies** and contextual circumstances.
    - Behavior outcomes: include participant behaviors required for the policy to have an effect, such as adherence, but can also refer to other behavioral outcomes occurring intentionally or unintentionally.
- **Health outcomes**: health-related behaviors, morbidity, mortality, well-being, and quality of life.
- **Non-health outcomes:** all other relevant impacts of an intervention, e.g. social, economic, or environmental outcomes
- **Unintended or harmful consequences** (including those due to **interactions** with **other policies**)
- ***Equity focus***: potential differential impacts of policy across population groups should be monitored

**CONTEXT** constitutes the geographical, epidemiological, socio-cultural, socio-economic, ethical, legal, and political context characteristics that act on local, national, and international levels.

**Context** also includes **other policies** occurring in a similar time frame as the policy of interest.

**CONTEXT**

**Policy formulation and design**

- **Problem definition**
- **Policy content** (i.e. policy design should preferably be evidence-based; involvement of the public of stakeholders (at micro, meso and macro level) important; policy re-design based on outcomes of implementation and impact evaluation; anticipate how policy of interest may **interact** with **other policies**)
- ***Equity focus*** (policy incorporates actions to reduce health inequities across population groups; policies targeting structural conditions are more likely to reduce inequities than those calling on individual agency)

**Implementation evaluation**

**CONTEXT**

**Impact evaluation**

**Appendix 5: Main feedback on the interim system-based PEN framework given by PEN researchers during an online workshop in January 2022**

- Equity is an overarching concept affecting all policy domains (just like Context), and could therefore be placed in the figure as separate construct, out of the content, impact and implementation boxes. However, it was decided to rather keep it in each box, as that allows specifying *how* equity is important for each of the domains.
- Most participants liked to keep relative lengthy texts within the figure instead of just mentioning key concepts in each of the boxes. The main reason was that concepts can have a different meaning to people from different fields, and therefore describing in more detail what is meant helps understanding what is shown in the figure.
- It was noted that little information was provided in the framework about what should actually be the content of policies, e.g. which policies should be implemented to promote PA and healthy diets. Some participants stressed that this was totally fine, since policy content is very much depended on the stakeholders involved in the discussion, what is seen as the problem, how the problem is defined and who pushes the problem definition, the context of the problem, etc. However, others indicated that mentioning existing frameworks as an example, for instance the Food-EPI framework and PA-EPI framework, which specify evidence-based policies for promoting PA and healthy diets (as that is what PEN is about), would help to show the complexity and variety of content that should be considered. We decided to mention this in the accompanying text.
- It was raised whether we wanted the framework to be more generic, and thus applicable on all kinds of policies that in some way affect health, or specific on policies promoting PA and healthy diets. We decided to keep the figure generic, but in the accompanying text, give PA- and diet-related examples (as these were the focus of PEN).
- The concepts mentioned in this framework should align with the Glossary paper that will also be published in the special issue, which presents all key concepts used in the PEN project.
- In the policy implementation box, it should be mentioned that policy processes, actions, as well as characteristics of the setting or context (including other policies) may act as barriers or facilitators of implementation.
- Re. policy implementation, it should be stated that policy comprises a package of instruments including communication, economy and regulation, and refer in the text to: Bemelmans-Videc M-L, Rist RC, Vedung EO. Carrots, Sticks & Sermons: Policy Instruments and Their Evaluation. Transaction Publishers, 2011.
- Regarding policy outcomes domain, include obesity as an example of a health outcome, and diet and PA as examples of health-related behaviours. Mention sustainability-related outcomes as an example of important non-health outcomes.
- Regarding policy outcomes domain, stress in the figure as well as the text that continuous monitoring and evaluation of multiple outcomes is needed (whereby findings feed back into policy formulation and implementation).
- Regarding policy outcomes domain, mention that a conceptual systems model should be developed to pre-specify how a policy is expected to affect outcomes in short, medium and long term, including feedback loops and interactions (including those between non-health and health outcomes)
- Regarding policy outcomes domain, mention the drawbacks of collecting individual level data vs. population level data.
- When it comes to stakeholders, mention that the target population is also seen as a stakeholder, together with e.g. implementers, policy makers, and lobbying groups.
- Mention the Multiple Streams model (Kingdon (1995)).
- In the Discussion section of the paper, mention how systems thinking has shifted our thinking of ‘environmental’ determinants of PA and diet more towards a broader perspective on how behaviours, and policies seeking to affect these, interact within food systems and transport systems.
- Regarding the influence of the context, mention PEN-specific examples, e.g. role of jurisdiction on EU level (PEN WP1), SUMPs implementation (WP6), SSBs tax implementation (WP6).
